# An Innovative Customized Biomimetic Hydrogel for Drug Screening Application Potential: Biocompatibility and Cell Invasion Ability

**DOI:** 10.3390/ijms23031488

**Published:** 2022-01-27

**Authors:** Keng-Liang Ou, Chiung-Fang Huang, Wen-Chien Lan, Bai-Hung Huang, Hsu-An Pan, Yung-Kang Shen, Takashi Saito, Hsin-Yu Tsai, Yung-Chieh Cho, Kuo-Sheng Hung, Hsin-Hua Chou

**Affiliations:** 1 Department of Dentistry, Taipei Medical University-Shuang Ho Hospital, New Taipei City 235, Taiwan; klou@tmu.edu.tw; 2 Taiwan Society of Blood Biomaterials, New Taipei City 221, Taiwan; 3 Division of Medicine for Function and Morphology of Sensor Organs, Department of Dentistry and Oral Surgery, Osaka Medical College, Osaka 569-8686, Japan; t-saito@hoku-iryo-u.ac.jp (T.S.); m225098012@tmu.edu.tw (H.-Y.T.); 4 Department of Oral Hygiene Care, Ching Kuo Institute of Management and Health, Keelung 203, Taiwan; jameslan@ems.cku.edu.tw; 5 Biomedical Technology R&D Center, China Medical University, Taichung 404, Taiwan; u109312001@cmu.edu.tw (B.-H.H.); andy.pan@3dglobalbiotech.com.tw (H.-A.P.); D204106003@tmu.edu.tw (Y.-C.C.); 6 3D Global Biotech Inc. (Spin-Off Company from Taipei Medical University), New Taipei City 221, Taiwan; 7 School of Dental Technology, College of Oral Medicine, Taipei Medical University, Taipei 110, Taiwan; chiung0102@tmu.edu.tw (C.-F.H.); ykshen@tmu.edu.tw (Y.-K.S.); 8 Department of Dentistry, Taipei Medical University Hospital, Taipei 110, Taiwan; 9 Graduate Institute of Dental Science, College of Dentistry, China Medical University, Taichung 404, Taiwan; 10 School of Dentistry, College of Oral Medicine, Taipei Medical University, Taipei 110, Taiwan; 11 Graduate Institute of Injury Prevention and Control, College of Public Health, Taipei Medical University, Taipei 110, Taiwan; 12 Department of Neurosurgery, Wan Fang Hospital, Taipei Medical University, Taipei 116, Taiwan; 13 School of Oral Hygiene, College of Oral Medicine, Taipei Medical University, Taipei 110, Taiwan; 14 Department of Dentistry, Wan Fang Hospital, Taipei Medical University, Taipei 116, Taiwan

**Keywords:** Pluronic F127, RGD peptide, spheroid, hydrogel, drug screening

## Abstract

The ability of Pluronic F127 (PF127) conjugated with tetrapeptide Gly-Arg-Gly-Asp (GRGD) as a sequence of Arg-Gly-Asp (RGD) peptide to form the investigated potential hydrogel (hereafter referred to as 3DG bioformer (3BE)) to produce spheroid, biocompatibility, and cell invasion ability, was assessed in this study. The fibroblast cell line (NIH 3T3), osteoblast cell line (MG-63), and human breast cancer cell line (MCF-7) were cultured in the 3BE hydrogel and commercial product (Matrigel) for comparison. The morphology of spheroid formation was evaluated via optical microscopy. The cell viability was observed through cell counting Kit-8 assay, and cell invasion was investigated via Boyden chamber assay. Analytical results indicated that 3BE exhibited lower spheroid formation than Matrigel. However, the 3BE appeared biocompatible to NIH 3T3, MG-63, and MCF-7 cells. Moreover, cell invasion ability and cell survival rate after invasion through the 3BE was displayed to be comparable to Matrigel. Thus, these findings demonstrate that the 3BE hydrogel has a great potential as an alternative to a three-dimensional cell culture for drug screening applications.

## 1. Introduction

Drug screening studies involve accurate knowledge or information about a disease, especially cancers that have a high recurrence rate and rapid metastasis, which can ultimately increase the effectiveness of drugs or therapies [1,2]. Spheroid cell culture is a three-dimensional (3D) culture system that resembles an in vivo microenvironment, which is capable of providing accurate knowledge or information about a disease which, in turn, can increase the effectiveness of drugs or therapies [3,4,5,6]. Therefore, the 3D culture system, which may be used useful in drug screening studies, is very effective. Hydrogels are often used as cell culture media because of their ability to form a 3D environment in vitro with superior chemical and biophysical properties, as well as being able to mimic environmental physiological bioactivity [4,7,8]. The commercial product Matrigel is one type of hydrogel that is recognized as the gold standard of the 3D microenvironment in cell cultures [4].

Pluronic F127 (PF127) is a synthetic triblock copolymer made of polyethylene oxide (PEO)–polypropylene oxide (PPO)–polyethylene oxide (PEO); its thermo-reversible gelation properties mean that it is extensively used in various biomedical applications, such as drug delivery vehicles and tissue engineering [9,10,11,12]. Historically, most synthetic polymers have lacked biological ligands, such as peptide, that can directly bind to cell surface receptors [13]. Therefore, Arg-Gly-Asp (RGD) peptide conjugated with polymer was reported to increase cell adhesion and proliferation [14,15,16,17]. In a previous study, PF127 and Gly-Arg-Gly-Asp (GRGD) peptide as a sequence RGD peptide were successfully prepared as 3BE hydrogel for in vitro cell culture application [9].

As stated above, the present study was aimed to evaluate PF127 conjugated with GRGD, hereinafter referred to as 3DG bioformer (3BE) hydrogel, in its ability as an alternative to the 3D microenvironment in cell culture, and thus, whether it can be implicated in drug screening in the future. For this purpose, a variety of cell types were used, including the MCF-7 breast cancer cell line as an invasive cell (often used as a model for drug screening with well-documented physiology in this study) [6,18], the MG-63 human osteosarcoma cell line as an invasive cell, and NIH 3T3 fibroblasts as normal cells, which were then cultured with 3BE, PF127, and Matrigel to assess the morphology of the formed spheroids, cell viability, and the ability of cells to invade the study medium.

## 2. Results

### 2.1. Spheroid Forming Evaluation

**Figure 1:**
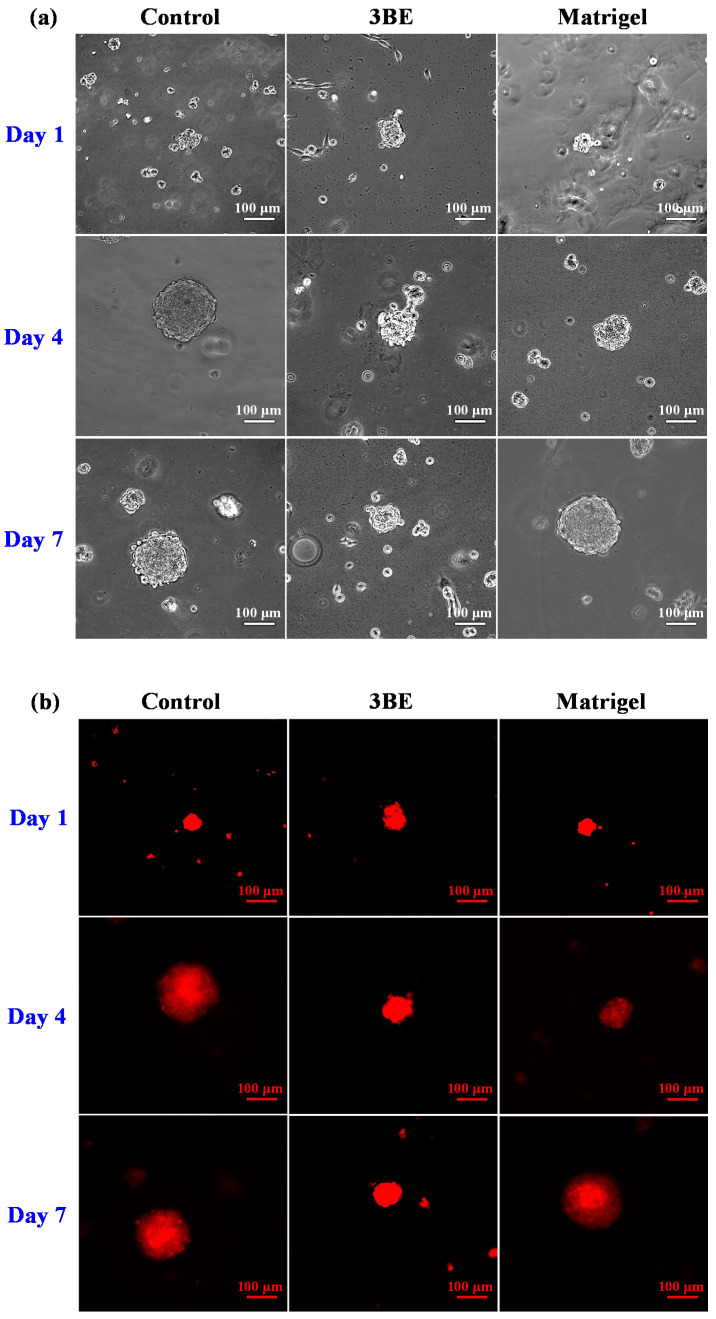
Morphology of NIH 3T3 fibroblast cell line in 3BE, Matrigel, and medium without gel (control) after 1 day, 4 days, and 7 days of culture: (**a**) bright-field images and (**b**) fluorescence images.

Figure 1 depicts the morphology features of NIH 3T3 fibroblast cell line through 3BE and Matrigel. The cells were aggregated and formed spheroid via 3BE and Matrigel, which connected singles cells together. Spheroids formed since the first day of culture on both 3BE and Matrigel. However, although 3BE was able to form spheroid cells, the growth was not as good as Matrigel after being cultured for seven days.

**Figure 2:**
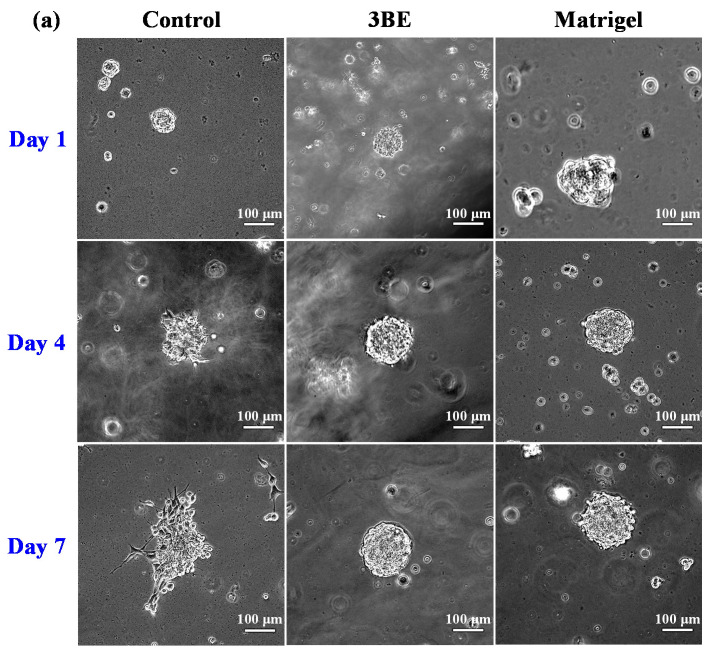

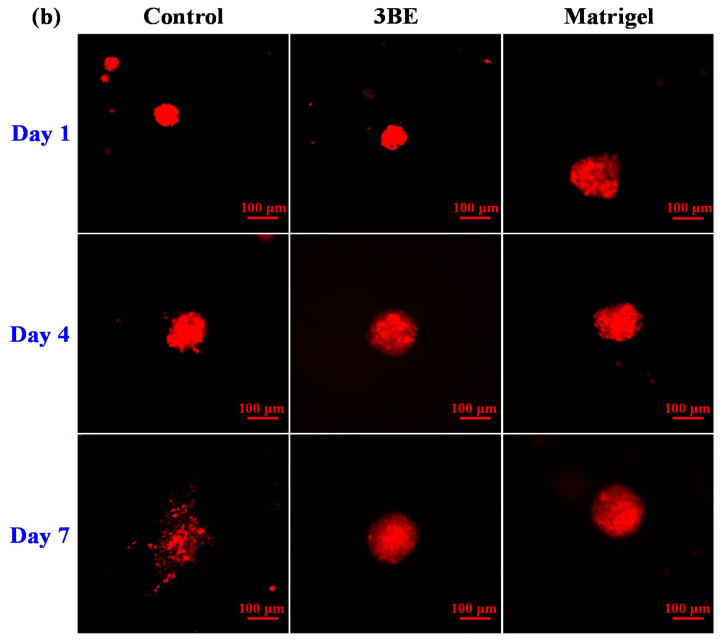
Morphology of MCF-7 cell in 3BE, Matrigel, and medium without gel (control) after 1 day, 4 days, and 7 days of culture: (**a**) bright-field images and (**b**) fluorescence images.

**Figure 3:**
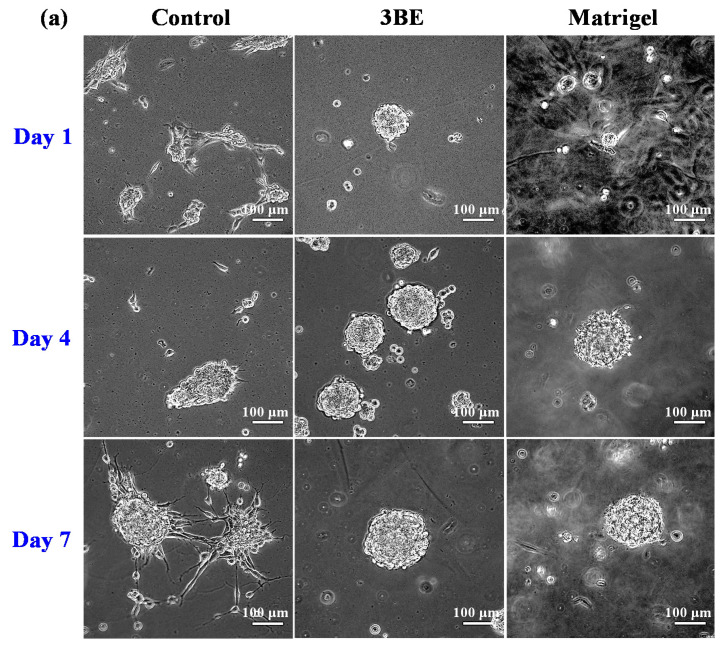

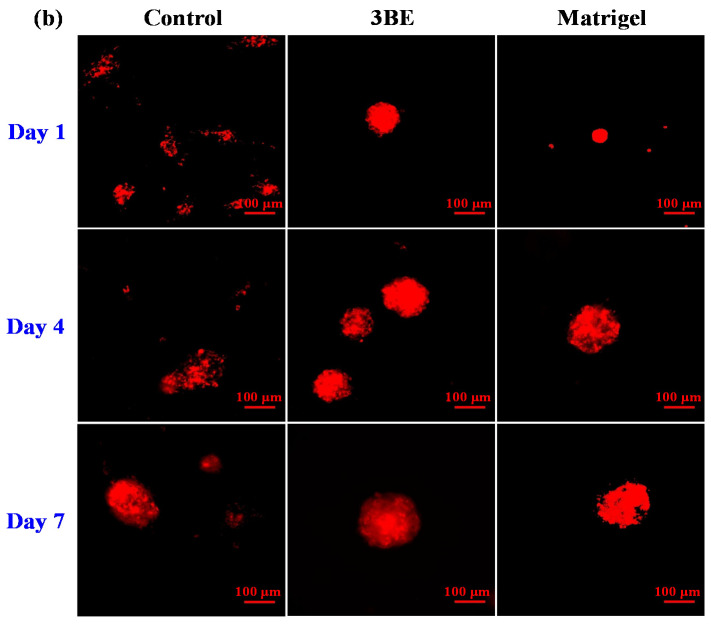
Morphology of MG-63 cell in 3BE, Matrigel, and medium without gel (control) after 1 day, 4 days, and 7 days of culture: (**a**) bright-field images and (**b**) fluorescence images.

Breast cancer cell lines and osteoblast cell lines cultured on 3BE and Matrigel were also shown to be capable of forming spheroids (Figure 2 and Figure 3). A few days after culture, some cells underwent aggregation to form increasingly solid spheroids. This phenomenon was seen in cells in 3BE and Matrigel; however, the spheroids in Matrigel were more solid than in 3BE, and spheroid growth in 3BE was not better than Matrigel.

### 2.2. Cell Invasion Evaluation

**Figure 4:**
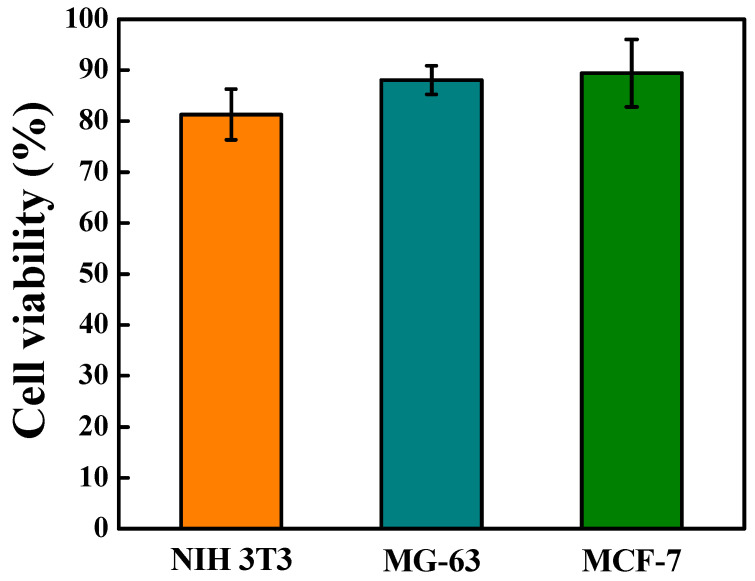
Cell viability of NIH 3T3, MG-63, MCF-7 cells after co-culture with 3BE. According to the ISO 10993-5, the tested sample is considered an acute cytotoxic potential when viability value of the tested sample is less than 70% of the medium only control (100%).

Cell Counting Kit-8 (CCK-8) results displayed the viability of NIH 3T3, MG-63, and MCF-7 after co-culture with 3BE, and which cell viability of these three different type cells were greater than 80% (Figure 4). According to the ISO 10993-5, there was an acceptable viability if the percentage cell vitality value was higher than 70%. Moreover, there were no statistically significant different cell viabilities of the NIH 3T3, MG-63, and MCF-7 cell lines after co-culture with 3BE.

**Figure 5:**
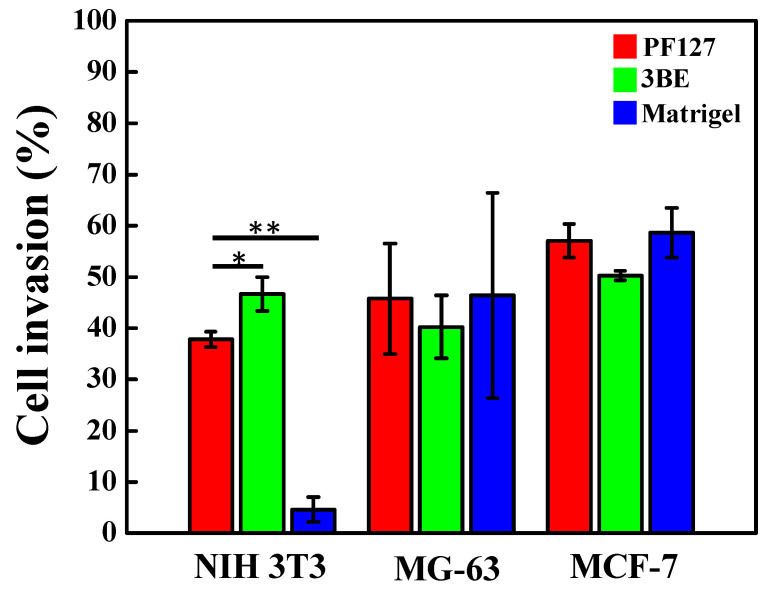
Cell invasion of NIH 3T3, MG-63, and MCF-7 cells in PF127, 3BE, and Matrigel (* *p* < 0.05 and ** *p* < 0.01). Only Transwell membrane (no gel coating) was used as a control group (denominator) for calculating cell invasion by Formula (1).

A cell invasion assay was required to confirm that the medium used had the ability to allow cells to invade, which indicated that the medium was more applicable. Figure 5 shows there were no statistical differences in the MCF-7 and MG-63 cell invasion ability in Matrigel, PF127, and 3BE. Nevertheless, NIH 3T3 cell invasion ability in 3BE was better than Matrigel. This result demonstrated NIH 3T3 in Matrigel has limited invasion ability. Globally, cells are able to invade through 3BE and analogue to Matrigel.

**Figure 6:**
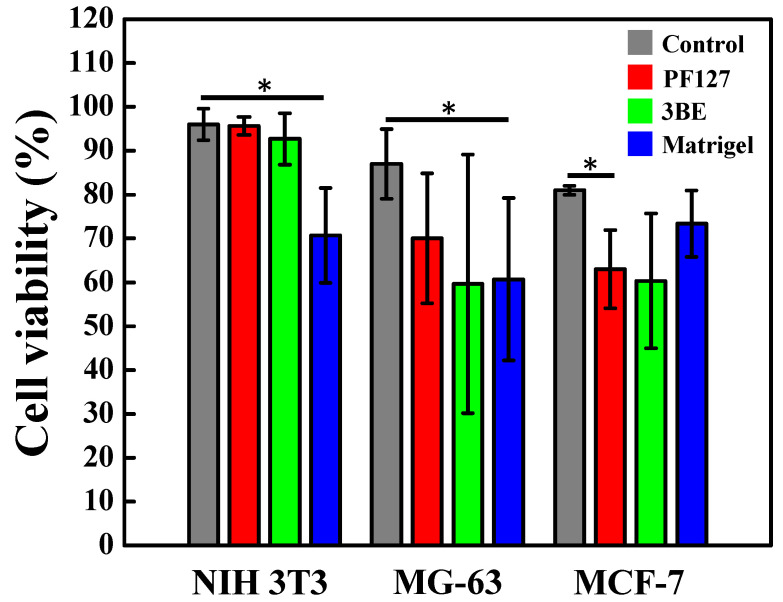
Cell viability of NIH 3T3, MG-63, and MCF-7 cells after invasion through PF127, 3BE, and Matrigel (* *p* < 0.05). Only Transwell membrane (no gel coating) was used as a control group. Cell viability was determined via Countess Automated Cell Counter after invasion.

After invasion via PF127, 3BE, and Matrigel, Figure 6 illustrates the viability of NIH 3T3, MG-63, and MCF-7 cells. The viability 3T3 cell after invasion through Matrigel was the lowest, whereas the viability of MG-63 and MCF-7 showed similar pattern after invasion through PF127, 3BE, and Matrigel, which had lower cell viability when invaded via 3BE. This pattern appears to be in line with the ability of cells to invade in PF127, 3BE, and Matrigel (Figure 4).

## 3. Discussion

This study used 3BE to evaluate its effectiveness as a 3D microenvironment in cell culture. Spheroids as 3D cells were formed by the aggregation of scattered cells due to the ability of the extracellular matrix (ECM) containing the RGD motif to bind to cell surface integrins, causing the expression of cadherin regulation, which accumulates on the surface of the cell membrane and holds bonds between surrounding cells, resulting in the formation of a spheroid [7,19,20,21]. During the formation of the spheroid, the spheroid will undergo various morphological changes. On the first day of culture, single cells aggregate with other cells. On days 2–3, the aggregating cells begin to join due to the encouragement of intercellular interactions. On days 3–5, multicellular aggregation forms dense spheroids with a smooth surface. Cell proliferation at the spheroid periphery causes the spheroid border to become irregular, which happens on days 5–7. Afterward, the size and morphology of the spheroid did not change significantly [6]. The spheroid formation in 3BE resembled in Matrigel, but the growth in 3BE was not as good as Matrigel as the gold standard. This may be due to differences in the composition of the medium [22]. Matrigel is rich in extracellular matrix protein and various growth factors, making it applicable, yet, it has the potential for xenogenic contamination because it is obtained from animals [23]. Therefore, 3BE can be used as an alternative 3D cell culture medium. Even the formation of spheroids on 3BE is as fast as Matrigel.

Moreover, the formation of spheroids on 3BE is possible because this medium resembles the ECM and contains various membrane proteins [7,9,24]. 3BE with GRGD peptide content can induce cells for self-assembly. This peptide motif can promote cell proliferation and differentiation [9]. However, RGD peptide is known to be sensitive to various types of cells including osteoblasts and fibroblasts, and can even cause cell death [24,25]. However, the use of RGD peptides in low concentrations allowed cell survival [24]. RGD peptide is also able to increase the attachment of fibroblast cells, but GRGD peptide as a sequence of RGD binds more to osteoblast cells [16,24]. Therefore, in this study, the viability of osteoblast cells was higher than that of fibroblasts cells. Regardless, this study showed that 3BE was not toxic to NIH 3T3, MG-63, and MCF-7.

Cell invasion is associated with cancer cell metastases, which refers to the migration of cells through the extracellular matrix [2,26,27]. Therefore, research on cell invasion is needed to obtain better anti-metastasis therapy [28]. In this study, NIH 3T3 cells were less able to invade through Matrigel because they were non-invasive or healthy cells [29]. The content of collagen and laminin in Matrigel is supposed to inhibit the penetration of benign cells and healthy cells, but if normal cells are impregnated with malignant cells, they will be able to penetrate Matrigel [30,31]. Overall, it was seen that 3BE was able to cause cells to migrate. Cell migration is altered by the biphasic hydrogel phenomenon associated with ligand density and cell adhesion, causing a traction force to be exerted on the substrate to move the cell [17,32]. It has been reported that focal adhesion affects cell migration due to mechanical stimuli obtained from the environment [33,34,35]. In this case, integrins as receptors will target the ECM by transmitting both outside-in signals and inside-out signals [33]. Other studies have shown that the use of RGD peptides has the potential to inhibit cell migration [36]. This can be explained because the cells have various integrins targeted for the ECM, and the cells can use numerous adhesion receptors to attach to the ECM [36]. This phenomenon explains the results of previous study, which showed lower cell migration ability in 3BE consisting of hydrogel PF127 with RGD peptide compared to PF127 alone without modification. However, the RGD peptide did not absolutely inhibit cell attachment and migration. Indeed, the remodeling of hydrogel PF127 with RGD peptide is a requisite for cell self-assembly in 3D cell culture [9]. As mentioned above, further investigations such as osmotic pressure, mechanical strength, rheological property, single-cell western blotting, and polymerase chain reaction must be carried out to provide scientific information concerning material properties and cellular reaction in the presence of potential 3BE hydrogel.

## 4. Materials and Methods

### 4.1. Materials Preparation

The present study used 3BE hydrogel that was synthesized from the polymer PF127 (Pluronic^®^ F127; Sigma 0.709 mmol, Taipei, Taiwan) and was remodeled by tetrapeptide Gly-Arg-Gly-Asp (GRGD; RDD Lab. Inc., New Taipei City, Taiwan) as described in a previous study [9]. Briefly, the triethylamine (TEA; Sigma 0.709 mmol, Taipei, Taiwan), 4-(dimethylamino)-pyridine (DMAP; Sigma 0.709 mmol, Taipei, Taiwan), and 4-methacryl oxyethyl trimellitic anhydride (4-META; Sigma 1.687 mmol, Taipei, Taiwan) were added in the dissolved solution of PF127, and the reaction mixture was stirred at 25 °C for 16 h under a nitrogen atmosphere. The precipitate generated by adding of diethyl ether (Sigma, Taipei, Taiwan) was filtered and dried with a high vacuum pressure to obtain the PF127-4-META. Subsequently, the 4-(dimethylamino) pyridine (Sigma, Taipei, Taiwan), *N*,*N′*-dicyclohexylcarbodiimide (Sigma 4.650 mmol, Taiwan), and N-hydroxysuccinimid (NHS Sigma 4.650 mmol, Taipei, Taiwan) were added in the dissolved PF127-4-META, and stirred at 25 °C for 16 h under a nitrogen atmosphere. Diethyl ether was then added to the reaction solution to form a precipitate. The residual solvent was removed to gain the PF127-4-META-NHS under a high vacuum pressure. After the synthesis of PF127-4-META-NHS, the triethylamine (Sigma 0.218 mmol, Taipei, Taiwan), PF127-4-META-NHS, and GRGD were added in a solution containing 2.6 mL *N*,*N*-dimethylformamide (DMF; Sigma, Taipei, Taiwan) and stirred at 25 °C for 16 h under a nitrogen atmosphere. In the next step, the reacted mixture solution was freeze-dried to remove the DMF, and then chilled methanol was added to create precipitate solution. Finally, the precipitate solution was filtered and dried to form the copolymer powder of PF127-4-META-GRGD (i.e., 3BE) under a high vacuum pressure.

### 4.2. Cell Culture

The fibroblasts cell line (NIH 3T3; ATCC-CRL-1658), osteoblast cell line (MG-63; ATCC-CRL1427), and human breast cancer line (MCF-7) were received from the Bioresource Collection and Research Center, Hsinchu, Taiwan. The cells were maintained using Eagle’s Minimum Essential Medium (MEM; Gibco, Thermo Fisher scientific, Waltham, MA, USA) and Roswell Park Memorial Institute Medium (RPMI 1640; Gibco, Thermo Fisher Scientific, Waltham, MA, USA), supplemented with 10% fetal bovine serum (FBS), 100 IU/mL penicillin, and 100 mg/mL streptomycin. The cells were cultured in a humidified incubator enriched with 5% CO_2_ at 37 °C and sub-cultured every 3 days.

All coating reagents were prepared as recommended by the manufacturers. The volume and concentration of the substances used for coating the wells were 1.0% Agarose (Sigma, Taipei, Taiwan). The coating reagents were then mixed with H_2_O to a total volume of 100 µL per well of a 96-well plate. Afterwards, the coated cultures were preincubated overnight at 4 °C. The coated wells were washed once with Dulbecco’s phosphate-buffered saline (DPBS), followed immediately by cell seeding. The volume of coating substances was adjusted according to the growth area when different culture vessels were used.

Cells were plated at a density of 10^3^ cells per each well into 96-multiwell plate for 24 h. One day after the initial plating, cells were treated and cultured in the same gel-diluted growth media containing PF127, 3BE and Matrigel. The gel-containing media was detected by Cell Counting Kit-8 (CCK-8) after 1 day, 4 days, and 7 days from the initial plating. The cells were cultured in gel-diluted growth media with 5% CO_2_ incubation at 37 °C during the whole test period. After gel treatment, 100 µL of CCK-8 (Sigma, Taipei, Taiwan) were added to every single well in the plate, which was incubated for 2 h at 37 °C. After the incubation, the absorbance was measured at 460 nm in a spectrophotometer (BioTek Epoch, Winooski, VT, USA). The morphology of the attached NIH 3T3 and MCF-7 cell on 3BE, Matrigel, and medium only as a control, was observed using a IX73 inverted microscope (Olympus, Tokyo, Japan).

### 4.3. Labeling Cells with Lipophilic Fluorescent Dyes

Prior to 3D cell culture, the cells were labelled with the fluorescent vital dye, PKH26 (General PKH26-GL cell linker kit, Sigma Aldrich, Taipei, Taiwan) according to the manufacturer’s instructions. The cell membrane of 10^3^ cells per mL were stained with the fluorescent marker and cultured in a 96-multiwell plate. After 1, 4 and 7 days of culture, the morphology of the attached NIH 3T3, MCF-7 and MG63 cells on 3BE, Matrigel, and medium only (blank) as a control was observed using the IX73 inverted microscope (Olympus, Tokyo, Japan) with an excitation wavelength of 543 nm, and an emission wavelength.

### 4.4. Cell Invasion Assay

**Figure 7:**
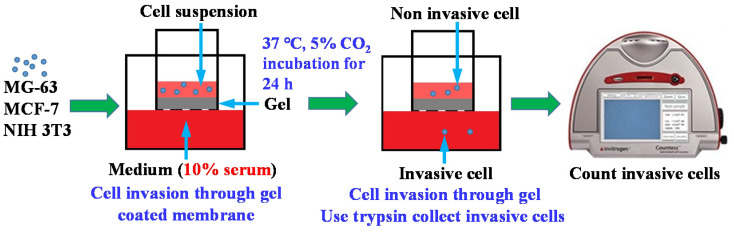
Schematic diagram of the Boyden chamber assay.

Cell invasion ability in PF127, 3BE, and Matrigel was assessed through the Boyden chamber assay, as shown in Figure 7.

The PF127, 3BE, and Matrigel was diluted in Dulbecco’s Modified Eagle Medium-High Glucose (DMEM-HG) without FBS to a final concentration of 1%. An 800 µL DMEM-HG medium with 10% FBS was added into 24-well plate. Then, 200 µL of cell samples and DMEM-HG (0% FBS) was carefully transferred to the center of each Transwell. Only Transwell membrane (no gel coating) was used as a control group. The NIH 3T3, MG-63, and MCF-7 cells were trypsinized and resuspended with DMEM-HG without FBS. Next, 200μL cell suspension was seeded into the upper chamber of each Transwell. The final cell density was 5 × 10^5^ cells/well. After incubation for 24 h, non-invasive cells were removed from the upper surface of the membrane. The NIH 3T3, MG-63, and MCF-7 cells were trypsinized and collected for cell counting analysis. Finally, Countess Automated Cell Counter (Invitrogen) was applied to count cell number and measure cell viability. The invasion percentage of the cell was calculated by following Formula (1):

(1) $$\%\;\mathrm{invasion}=\frac{\mathrm{cell}\;\mathrm{number}\;\mathrm{invasion}\;\mathrm{through}\;\mathrm{the}\;\mathrm{gel}}{\mathrm{control}\;\mathrm{cell}\;\mathrm{number}}\times 100$$

### 4.5. Statistical Analysis

The experimental data were analyzed via SPSS statistic software (Version 19.0., SPSS Inc., Chicago, IL, USA). The difference between multiple groups were determined by one-way analysis of variance followed by Tukey’s HSD post-hoc test. The *p* value less than 0.05 was indicated as statistically significant.

## 5. Conclusions

The 3BE presented as slightly inferior to Matrigel in forming spheroid cells, although it had good biocompatibility with NIH 3T3, MG-63, and MCF-7 cells. The performance of cell invasion and the survival rate after cell invasion through 3BE were comparable to Matrigel. Therefore, this study suggests that 3BE mimics the natural ECM and produces cell invasion behavior, which can be applied for drug screening applications.

## Data Availability

Data is contained within the article.
